# Ndc80 Loop as a protein-protein interaction motif

**DOI:** 10.1186/1747-1028-8-2

**Published:** 2013-03-15

**Authors:** Ngang Heok Tang, Takashi Toda

**Affiliations:** 1 Laboratory of Cell Regulation, Cancer Research UK, London Research Institute, Lincoln’s Inn Fields Laboratories, 44 Lincoln’s Inn Fields, London, WC2A 3LY, UK

**Keywords:** Kinetochore, Ndc80/Hec1 complex, Spindle microtubule, Loop, Cdt1, Dam1 complex, Ska complex, TACC-TOG, Humans, Yeast

## Abstract

Our understanding of the structure and function of kinetochores has advanced dramatically over the past 10 years, yet how the plus end of spindle microtubules interacts with the kinetochore and establishes amphitelic attachment for proper sister chromatid segregation remains unresolved. However, several recent reports from different organisms have shed new light on this issue. A key player in microtubule-kinetochore interaction is the conserved Ndc80 outer kinetochore complex. In both yeast and human cells in particular, a ubiquitous internal ‘loop’ found in the Ndc80 molecule interrupting its C-terminal coiled-coil domain plays critical roles in protein-protein interaction, by recruiting microtubule-binding proteins to ensure proper kinetochore-microtubule attachment. In this commentary, we summarise the recent progress made and discuss the evolutionary significance of this loop’s role in microtubule dynamics at the kinetochore for accurate chromosome segregation.

## Background

During mitosis, the kinetochore complex attaches to the mitotic spindle to ensure accurate chromosome segregation [1-3]. The Ndc80/Hec1 complex, which is part of the KMN network (the KNL1/Mis12 complex/Ndc80 complex), is composed of a heterotetramer consisting of Ndc80/Hec1, Nuf2, Spc24 and Spc25 [1,4]. This complex is situated in the outer kinetochore and directly interacts with the microtubule [2,5]. However until recently, how the kinetochore establishes stable amphitelic attachment to the dynamic plus end of the spindle microtubule during mitotic progression remained largely unknown.

## Main text and discussion

### The Ndc80 loop and its binding partners

Structural studies have revealed that the Ndc80 complex has a dumbbell-like architecture consisting of an internal rod-shaped coiled-coil structure and a globular domain on each end [4,5]. In addition, a ubiquitous loop exists that interrupts the middle of the coiled coil domains. This loop was suggested to play a structural role in kinetochore geometry and/or the tension-sensing mechanism, yet its precise function at the molecular level remained elusive for some time [6]. Recent studies from several laboratories have started to uncover its precise role [7,8].

**Figure 1:**
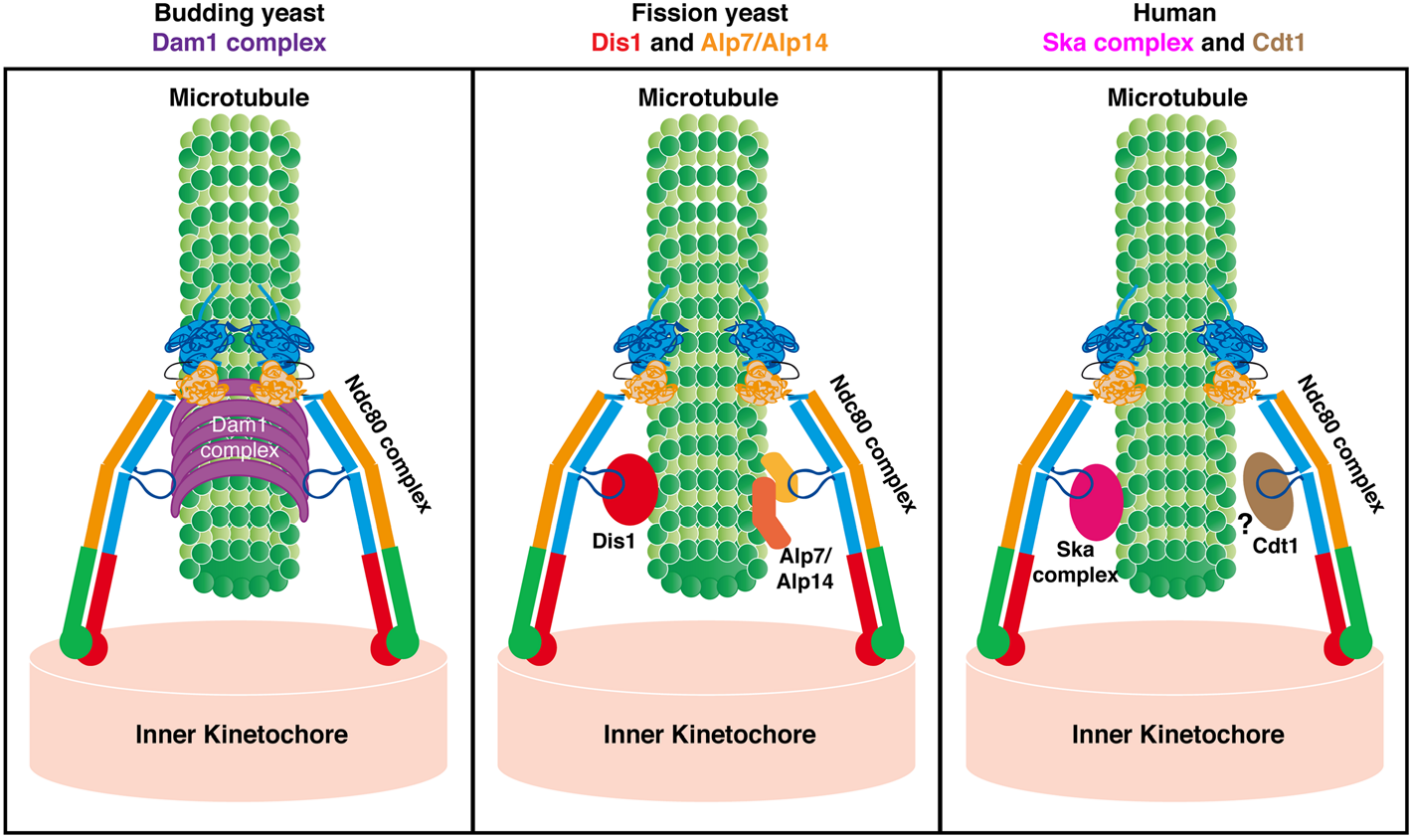
**The Ndc80 internal loop plays distinct roles in kinetochore-microtubule attachment by recruiting multiple regulatory factors to the outer kinetochore.** In budding yeast, the Ndc80 loop helps to recruit the Dam1 complex to the outer kinetochore (left); whilst in fission yeast, the Ndc80 loop binds to and recruits the Dis1/TOG and Alp7/TACC-Alp14/TOG complexes (centre). In higher eukaryotes, the Ndc80 loop has been shown to be able to recruit the Cdt1 protein and the Ska complex, either directly or indirectly (right). Note that no microtubule-binding activity has been reported for the Cdt1 protein (depicted by ?). We suggest that the Ndc80 loop plays a role in protein-protein interaction to ensure proper kinetochore-microtubule attachment.

By using specific *ndc80* mutants that contain mutations within the loop in *Saccharomyces cerevisiae*, the Tanaka lab showed that this region facilitates binding of the Dam1 kinetochore complex to the Ndc80 complex (Figure 1, left). This binding is essential for the conversion of lateral microtubule binding to end-on binding, which is a prerequisite for proper chromosome segregation [9]. Two other studies using mammalian (HeLa) cell lines unveiled the roles of the Ndc80 internal loop in recruiting the Ska (Ska1) kinetochore complex and the Cdt1 replication licensing protein (Figure 1, right) [10,11]; The Nilsson lab showed that in cells with the Ndc80 loop deleted or artificially reversed, lateral attachment could be formed, but proper end-on attachment failed to be established. This defect seemed largely due to the displacement of the Ska complex from the kinetochore [11]. On the other hand, the Cook and Salmon labs used a clever synchronise-release technique to uncover novel roles of the Cdt1 protein in mitotic progression upon the completion of S phase [10]. Intriguingly, Cdt1 localises to the kinetochore during early mitosis and appears to directly interact with the Ndc80 loop based upon various binding analyses. Unlike the Dam1 and Ska complexes however, no microtubule-binding activity has been shown for the Cdt1 protein. This makes it intriguing to look for possible microtubule associated proteins (MAPs) that Cdt1 interacts with on the kinetochores.

### Sequential binding of two TOG-TACC microtubule-associated proteins to the fission yeast Ndc80 loop

Our recent studies in *Schizosaccharomyces pombe* have uncovered that the Ndc80 internal loop binds to the Dis1/XMAP215/TOG and Alp7/TACC-Alp14/TOG MAP complexes (Figure 1, centre) [12,13]. In the *ndc80-21* mutant (in which a mutation in the loop region, L405P, is largely responsible for the phenotype), the Dis1 protein delocalises from the kinetochore, resulting in an unstable mitotic spindle and early mitotic arrest with no stable kinetochore-microtubule attachment being observed in this mutant [13]. Interestingly, we identified another *ndc80* loop mutant with a different mutation site in the loop (F420S), that showed normal Dis1 protein localisation to the kinetochore and normal mitotic spindle structure. In this mutant however, the Alp7-Alp14 complex was specifically delocalised from the kinetochore, leading to chromosome missegregation during anaphase A [12]. We speculate that in fission yeast, via the interaction with two different TOG (and TACC) proteins, the Ndc80 loop plays sequential roles in ensuring proper kinetochore-microtubule attachment and chromosome segregation. Binding of the Dis1 protein to the Ndc80 loop in early mitosis, which is independent of microtubules [14], is important for mitotic spindle assembly and stabilisation, whilst subsequent binding of the Alp7-Alp14 complex, which requires spindle microtubules [15], ensures proper anaphase progression.

### Roles of the Ndc80 loop in microtubule dynamics at the kinetochore

Microtubules are intrinsically dynamic and bind to a cohort of MAPs. In particular, the mitotic spindle changes its polymerising/depolymerising activity throughout the different stages of mitosis. This leads us to suggest that the Ndc80 internal loop, by interacting with different proteins during different stages, helps to regulate microtubule dynamics. It would be interesting to test this idea by reconstituting each protein complex in the presence of microtubules *in vitro*.

To date, all the Ndc80 loop-interacting proteins identified have no reported microtubule depolymerising activity. Although the Dam1 and Ska complexes have been shown to track on the plus end of depolymerising microtubules and the TOG proteins are in fact microtubule polymerases, it remains enigmatic as to how microtubule depolymerisation per se is directionally driven during anaphase A [16,17]. It should also be of note that whether the Dam1 and the Ska complexes interact with the loop remains to be established [16,18]. Therefore, how the kinetochores remain attached to the depolymerising microtubule plus-ends during anaphase remains a major question to be answered.

## Conclusions

Although the proteins bound to the Ndc80 loop in different organisms show no sequence homology to one another, these studies showed the importance and capabilities of the Ndc80 internal loop region as a protein-protein interaction motif, thereby ensuring proper chromosome segregation, and that mitotic progression is conserved from yeasts to higher eukaryotes. Furthermore, these studies provided further insight into how the kinetochore, in particular the Ndc80 complex, regulates microtubule dynamics during mitosis.

## Abbreviations

MAP: Microtubule-associated protein; TACC: Transforming acidic coiled coil; +TIP: Plus-end tracking protein; TOG: Tumor overexpressed gene

## Competing interests

The authors declare that they have no competing interests.

## Authors’ contributions

NHT and TT jointly wrote the paper. Both authors read and approved the final manuscript.
